# Contrasting Influences of Geographic Range and Distribution of Populations on Patterns of Genetic Diversity in Two Sympatric Pilbara Acacias

**DOI:** 10.1371/journal.pone.0163995

**Published:** 2016-10-21

**Authors:** E. Levy, M. Byrne, D. J. Coates, B. M. Macdonald, S. McArthur, S. van Leeuwen

**Affiliations:** Science and Conservation Division, Department of Parks and Wildlife, Perth, Western Australia, Australia; National Cheng Kung University, TAIWAN

## Abstract

The influence of geographic range on species persistence has long been of interest and there is a need for a better understanding of the genetic consequences for species with restricted distributions, particularly with the increasing rate of global species extinctions. However, the genetic effects of restricted range are often confounded by the impacts of population distribution. We compared chloroplast and nuclear genetic diversity and differentiation in two acacias, the restricted, patchily distributed *Acacia atkinsiana* and the widespread, semi-continuously distributed *A*. *ancistrocarpa*. Lower intra-population diversity and higher differentiation between populations were seen in *A*. *atkinsiana* compared to its widespread congener, *A*. *ancistrocarpa*. There was little evidence of geographical influences on population genetic structure in *A*. *ancistrocarpa* whereas *A*. *atkinsiana* exhibited nuclear genetic structure with isolation by distance, differentiation of near-coastal populations from those in the ranges, and differentiation of peripheral populations from those in the centre of the distribution. These results are consistent with expectations of the effect of geographic range and population distribution on genetic diversity, but indicate that distribution of populations rather than geographic range has influenced the observed genetic structure. The contrasting patterns observed here demonstrate that conservation approaches for species management and ecological restoration need to consider the distribution of populations in geographically restricted species.

## Introduction

Geographic range is considered to be one of a number of key attributes of plant species that can indicate increased risk of extinction [1–4]. Species occupying narrow or restricted ranges are more vulnerable to environmental stochasticity and genetic declines than widespread species [5–9]. There has been a strong focus in the literature on examining levels of genetic variation within and between populations of geographically restricted plants, often in comparison to common or widespread congeners [10, 11–16]. Such an approach is valuable as it reduces the confounding effect of phylogenetic differences among species on genetic patterns [7, 17].

In line with population genetic theory, broad comparisons between geographically restricted and widespread species have indicated that in general, geographically localised species tend to have less genetic variation than common, widespread species [6, 7, 18, 19]. However, there are notable contradictions to this trend with a number of studies finding similar or greater levels of genetic variation in localised plants compared to widespread congeners [12, 13, 16, 20, 21]. Geographically restricted species are also expected to have less genetic divergence between populations than their widespread counterparts because populations of widespread species will, on average, be further apart from one another across the entire species range than those of restricted species and under an isolation-by-distance model, genetic divergence is predicted to be greater in widespread species [19, 22]. However, there are no strong overall trends in the pattern of population genetic differentiation and structure among populations of geographically localised plants compared to widespread congeners [6, 18, 19, 23]. These inconsistencies could be due to the influence of numerous factors, including mating systems, life history traits, chromosomal variation, population distribution and other ecological traits related to gene flow [23–25].

The distribution of populations is one factor that is likely to have a significant impact on patterns of genetic diversity both within and between populations. Patchily distributed populations are often smaller than semi-continuous populations, making them more vulnerable to the effects of genetic drift and inbreeding that deplete genetic variation [5, 26–28]. Further, patchily distributed populations are expected to exchange fewer genes than semi-continuous populations, thus reducing genetic variation within populations and increasing genetic divergence among populations [5, 26, 28]. The effects of population distribution on gene flow and genetic variation have been well documented and generally find fragmented populations have low genetic variation and high genetic divergence between populations [29–36]. However, the influence of distribution of populations on genetic variation and structure is often overlooked in comparisons of widespread and restricted species and, in many studies, the distribution of populations is not described. Therefore, it is difficult to assess genetic effects due to range and those due to differences in distributions of populations.

The broad Pilbara region of north-western Australia provides an ideal opportunity to examine the influence of geographic range and distribution of populations on historical and contemporary patterns of genetic structure and intra-population genetic variation in plants. *Acacia* is a dominant genus in Australian environments, particularly in arid ecosystems such as the Pilbara which is a centre of endemism for *Acacia* [37], with many combinations of endemic species and their widespread congeners. The Pilbara region has had a stable geomorphological history and is characterised by the ancient geological formations of the Chichester and Hamersley Ranges surrounded by flatter areas along the coast to the west, and sandy deserts beyond the region to the east. While the geomorphology of the region is well understood [38], knowledge of the genetic diversity of the flora and fauna of the Pilbara is more limited, although studies in animals have shown a combination of high localised endemism and high species diversity [39–41]associated with the physiogeography and underlying geology.

There have been several assessments of genetic structure in Pilbara invertebrates [42–45] and vertebrates [41, 46–48], but plants have received significantly less attention [49, 50]. The few genetic studies on plants show that Pilbara populations are distinct from non-Pilbara populations but within the Pilbara, genetic structure is limited [49–51]. However, there is evidence for upland ranges being refugia. Sakaguchi *et al* [50] suggest the low genetic variation and distinct genetic lineages of *Callitris glaucophylla* in the Pilbara compared to other areas, are evidence of confinement to microrefugia within the region during multiple glacial cycles. A phylogeographic study of *Eucalyptus leucophloia* showed genetic signature of the Hamersley Range being a refugium during arid climatic cycles with later expansion into non-range areas [51]. Analysis of evolutionary history in the Australian arid zone shows major species radiations in response to development of arid environments through the Pliocene, with lineage divergence within species developing through the climatic cycles of the Pleistocene [52]. Within the Pilbara, persistence of species in refugia is the likely response of the biota to the climatic oscillations of the Pleistocene, as ranges have been hypothesised to provide refugial opportunities for biota, and thermal buffering from the ocean may have facilitated persistence of species in coastal areas [52]. Species can be influenced by different historical processes so assessment of historical influences on genetic diversity through analysis of chloroplast diversity is also important to provide a basis for understanding contemporary influences on patterns of diversity.

Here, we investigated patterns of genetic diversity and structure in two Pilbara acacias that differ in their range and in the distribution of their populations. *Acacia atkinsiana* has a restricted distribution and is endemic to the Pilbara region compared to its widespread congener *A*. *ancistrocarpa* that is distributed across northern Australia. The restricted *Acacia atkinsiana* forms dense stands but has a patchy distribution compared to the semi-continuous distribution found in the widespread *A*. *ancistrocarpa* [53]. Although their geographic range and population distribution differ, the reproductive biology of both species is similar, in particular, seed size is also similar in both species and there are no key differences in their pollen and seed dispersal. Acacias tend to be generalist insect pollinated [54] and neither species possesses any obvious modifications for other types of dispersal, for instance, wind or animal dispersal. *Acacia ancistrocarpa* has explosive dehiscence of its seed pods, although seeds are only propelled a very short distance from the plant, with most remaining directly beneath the plant. Therefore, we are able to make a comparison of species with differing geographical distributions and population distributions without the confounding effects of differences in gene flow. We assessed genetic diversity and structure using both nuclear and chloroplast markers to provide complementary understanding of factors influencing contemporary and historical variation within the species. Specifically, we investigated whether the distribution of genetic variation within and among populations of *A*. *atkinsiana* and *A*. *ancistrocarpa* differed, and whether these differences were associated with geographic range or distribution of populations. Consistent with population genetic theory, we predicted that genetic variation would be lower in the geographically restricted and patchily distributed *A*. *atkinsiana* than in the widespread and semi-continuously distributed *A*. *ancistrocarpa*. Further, the contrasting population distributions of the species led us to hypothesise that the patchily distributed *A*. *atkinsiana* would have greater genetic structure than the semi-continuously distributed *A*. *ancistrocarpa*, despite its narrower geographic range.

## Methods

### Study species

**Fig 1:**
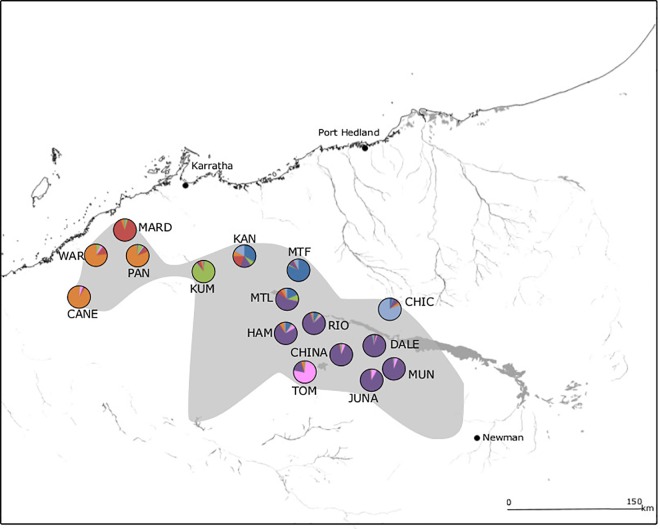
Geographical distribution of populations and inferred nuclear genetic clusters from TESS analysis for *Acacia atkinsiana*. The species distribution is shown in grey. Each colour represents a distinct genetic cluster and the size of each section indicates the proportion of each individual’s genome assigned to each cluster, averaged across the population. Major water courses and the Fortescue floodplain are shown.

**Fig 2:**
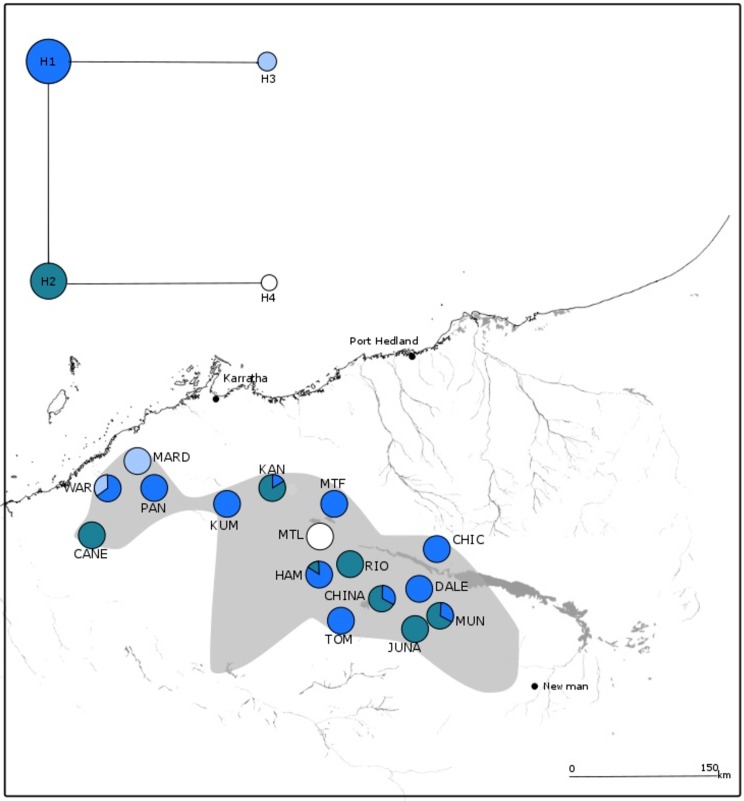
Geographical distribution of populations and chloroplast haplotypes for *Acacia ancistrocarpa*. The species distribution is shown in grey. All chloroplast haplotypes found in each population are shown with section sizes indicating the proportion of individuals with that haplotype. The haplotype network is shown in the upper left corner, each circle represents a distinct haplotype and its size indicates its relative frequency amongst all individuals. Branch length indicates the number of mutational steps between haplotypes and unobserved haplotypes are represented by black circles. Major water courses and the Fortescue floodplain are shown.

*Acacia atkinsiana* is endemic to the Pilbara and adjacent Ashburton bioregions, being distributed mainly on the southern Roebourne Plain and in the Hamersley and Chichester Ranges [53] (Fig 1), whereas *A*. *ancistrocarpa* is widespread across northern Australia, including in the western, central and eastern Pilbara and the Hamersley and Chichester Ranges [53] (Fig 2). *Acacia atkinsiana* is usually found in pure stands on water gaining sites, rocky, loamy, stony ground or ironstone hills. It is killed by fire, regenerating from soil-stored seed and there is no evidence of hybridisation with other acacias [55]. In contrast, *A*. *ancistrocarpa* grows mostly on acidic red sandy soils and stony pediments, often near water courses or water gaining sites. It is known to hybridise with *A*. *stellaticeps*, *A*. *citrinoviridis*, *A*. *arida* and *A*. *orthocarpa*. Hybrids with the latter three species are rare, and in the case of *A*. *orthocarpa*, are sterile [53]. *Acacia ancistrocarpa* is also generally killed by fire but does have the ability to coppice from root stock. Acacias tend to be generalist insect pollinated and seed dispersal is commonly facilitated by ants or along waterways [54, 56]. *Acacia atkinsiana* and *A*. *ancistrocarpa* are classified to the same taxonomic section (Juliflorae) and their relationship can be seen in a detailed phylogeny of Acacia (http://phylolink.ala.org.au/phylo/getTree?studyId=92#node/395373a92f9db36c18fc0845ebcf9db5).

Pure (non-hybrid) populations of *A*. *atkinsiana* and *A*. *ancistrocarpa* were sampled within each species’ distributional range across the Pilbara (Figs 1 and 2). Reflective of a larger distribution within the Pilbara, distances between sampled populations of *A*. *ancistrocarpa* were greater (mean distance between sampled populations: 263km (range: 39km-629km)) than *A*. *atkinsiana* (mean distance between sampled populations: 147km (range: 30km-336km)). Phyllode material was collected from 24 plants within each of 16 populations of *A*. *atkinsiana* (384 plants) and 21 populations of *A*. *ancistrocarpa* (504 plants). Permission for field studies was obtained from The Department of Parks and Wildlife. The field studies did not include any endangered or threatened species.

### DNA extraction and genotyping

DNA was extracted from *A*. *atkinsiana* and samples were genotyped at 11 microsatellite loci according to Levy *et al*. [57]. A Qiagen DNA PlantMini Kit was used to extract DNA from *A*. *ancistrocarpa* and samples were genotyped using seven microsatellite primers previously developed for acacias (AH16, 69, 29 [58], AM136, 30, 503, 400 [59]) and four loci developed for *A*. *atkinsiana* (AAB 11, 15, 19 and 26 [57]). Amplification reactions were carried out using 1.5–2.0 mM MgCl_2_ with 1M Betaine and cycling conditions of 96°C for 2 min; 30 cycles of 94°C for 30 s, 56°C for 30 s, 72°C for 30 s; and 72°C for 5 min. Reactions were run on an Applied Biosystems 3730 capillary sequencer and Genemapper 4.0 (Applied Biosystems, Foster City, California) was used to score alleles.

### Chloroplast sequencing

Non-coding spacer regions known to detect intraspecific variation in the chloroplast genome of Australian species [60] were trialed. The three most variable regions (trnV-ndhC, trnS-trnG5’2S [61] and D-loop atpF [62]) were selected for analysis of six individuals from each population, in each species, according to Byrne & Hankinson [60]. Amplification products were purified using AgencourtAMPure XP magnetic beads according to the manufacturer’s instructions and sequenced by Macrogen Inc using the EZSeq service.

Sequence data was examined for quality using ABI Sequence Scanner v1.0 and edited using Sequencher v5.0 (Gene Codes Corporation, Ann Arbor, MI USA http://www.genecodes.com); where possible, forward and reverse sequence data were assembled to create a contiguous sequence. The edited sequences for each chloroplast region were aligned using Clustal W 1.4 [63] and trimmed to equal lengths. A concatenated sequence file from the three variable chloroplast regions was assembled. Single base indels that followed an AT rich region were discounted and all other indels were coded as single characters.

### Data analysis

#### Nuclear diversity and population structure

The program FreeNa [64] was used to estimate null allele frequencies for each locus, based on the expectation maximization algorithm [65]. This program creates a data set corrected for null alleles and uses it to calculate global and pairwise *F*_*ST*_ values across all loci and for each locus. As there was little difference (*A*. *atkinsiana*: corrected F_ST_ = 0.182, 95% CI = 0.136–0.246, uncorrected *F*_*ST*_ = 0.186, 95% CI = 0.139–0.250; *A*. *ancistrocarpa*: corrected *F*_*ST*_ = 0.045, 95% CI = 0.024–0.079, uncorrected *F*_*ST*_ = 0.046, 95% CI = 0.023–0.085) between the corrected and uncorrected *F*_*ST*_ values, the original data sets were used for all remaining analyses.

For each species, microsatellite variation within each population was measured using allele frequency data, from which average allelic richness (AR), inbreeding coefficient (*F*_*IS*_), Shannon diversity index (I), observed (H_O_) and expected (H_E_) heterozygosity were calculated. Estimates of genetic diversity within populations and whether these differed between the two species, departures from Hardy Weinberg Equilibrium (HWE), and linkage disequilibrium (LD) were calculated using FSTAT [66]. GenAlEx 6 [67] was used to calculate Shannon diversity index. The selfing rate for each population was calculated using the robust multilocus method of David *et al*. [68].

Genetic divergence between populations was estimated by calculating pairwise *F*_*ST*_ values in FSTAT [66]. To test for patterns of isolation by distance (IBD) an *F*_*ST*_/(1–*F*_*ST*_) matrix was compared with a geographical distance matrix (log km) [69], using a Mantel test (10,000 permutations), calculated with the software package VEGAN v1.17–9 [70].

Population structure was analysed using an individual based Bayesian assignment approach implemented in TESS v2.3.1 [71]. This program groups individuals into the most likely number of clusters (*K*_MAX_) that maximises the within cluster Hardy-Weinberg and linkage equilibria. TESS incorporates spatial information and accounts for spatial autocorrelation (IBD). The admixture model in this program enables a proportionate membership of each individual to be assigned to each cluster. For each species, TESS was run with admixture (CAR model) using 50,000 sweeps following a burnin of 10,000 for 100 replicate runs of *K* ranging from 2–16 (*A*. *atkinsiana*) or 2–21 (*A*. *ancistrocarpa*) and applying a spatial interaction factor of 0.6. For each model, the Deviance Information Criterion (DIC) was obtained for each run and, for each value of *K*_MAX_, the mean DIC was plotted against *K*_MAX_. The best estimate of *K*_MAX_ was taken as the beginning of the plateau [72] and CLUMMP [73] was used to average the estimated membership probabilities for the 10% of runs with the lowest DIC.

#### Chloroplast haplotype diversity and phylogeographic structure

For each species, ordered and unordered within-population diversity (*v*_S_, *h*_S_), total diversity (*v*_T_, *h*_T_) and population differentiation (*N*_ST_, *G*_ST_) were calculated using PERMUT v2.0 [74]. Nucleotide diversity (*π*) was calculated in DnaSP v5.10.01 [75]. The likelihood of departures from neutrality and rapid population growth for each population were assessed using Tajima’s [76] *D* statistic, as implemented in ARLEQUIN v3.1 [77].

The presence of phylogeographical structure was assessed by testing if *N*_ST_ was significantly larger than *G*_ST_ using PERMUT [74] with 1000 permutations. We created a median-joining maximum parsimony network in NETWORK v4.6.1.1 [78].

## Results

### Nuclear diversity and differentiation

Most estimates of microsatellite variation were significantly lower in *A*. *atkinsiana* than in *A*. *ancistrocarpa*, except for observed heterozygosity which was similar between the two species (Table 1). The inbreeding coefficient (*F*_*IS*_*)* was more varied in *A*. *atkinsiana*, although departures from HWE were only detected in *A*. *ancistrocarpa* (10% of all possible locus/population combinations). These departures from HWE are most likely due to artefacts such as null alleles rather than biological causes such as inbreeding, as none of the selfing rates calculated using RMES were significantly different to zero. High (above 10%) null allele frequencies were present in all populations for locus AM503, but because AM503 was also the most variable and thus informative locus, and because removal of these null alleles did not alter population differentiation (*F*_*ST*_), we retained this locus for all analyses. There was no evidence of LD between any loci in any of the populations examined.

**Table 1:** Nuclear genetic diversity characteristics of *Acacia atkinsiana* and *A*. *ancistrocarpa* populations. Allelic richness (AR), observed heterozygosity (H_O_), expected heterozygosity (H_E_), inbreeding coefficients (F_IS_) and Shannon diversity indices (I) are means across all loci. Significant departures from HWE are indicated by an asterisk. Selfing rates(*s*) obtained using the robust multilocus method. *P* values relate to differences between species for each characteristic.

Species / Population	AR	H_E_	H_O_	F_IS_	I	*s*
***A*. *atkinsiana***						
CANE	3.3	0.419	0.436	-0.049	0.760	0.000
CHIC	2.7	0.460	0.394	0.133	0.711	0.226
CHINA	2.8	0.422	0.371	0.103	0.701	0.000
DALE	2.9	0.428	0.458	-0.071	0.716	0.103
HAM	3.2	0.497	0.511	-0.042	0.849	0.042
JUNA	2.4	0.393	0.367	0.066	0.617	0.000
KAN	3.8	0.534	0.500	0.040	0.952	0.032
KUM	2.8	0.264	0.284	-0.081	0.484	0.000
MARD	2.2	0.229	0.284	-0.265	0.389	0.005
MTF	3.3	0.522	0.515	0.010	0.877	0.012
MTL	3.3	0.504	0.492	0.011	0.857	0.000
MUN	3.0	0.407	0.390	0.026	0.697	0.000
PAN	3.2	0.429	0.466	-0.087	0.766	0.111
RIO	3.1	0.493	0.534	-0.083	0.837	0.000
TOM	3.5	0.416	0.375	0.091	0.757	0.177
WAR	3.2	0.403	0.508	-0.270	0.710	0.000
*Mean*	*3*.*0*	*0*.*426*	*0*.*430*	*-0*.*029*	*0*.*730*	*0*.*044*
***A*. *ancistrocarpa***						
CAR	5.6	0.548	0.462	0.154*	1.163	0.093
DAW	5.8	0.511	0.419	0.175*	1.103	0.000
FVA	5.0	0.531	0.470	0.115	1.071	0.000
HAM	5.3	0.487	0.402	0.172*	1.022	0.000
HDO	5.3	0.506	0.413	0.184*	1.048	0.122
IND	5.7	0.543	0.477	0.122	1.148	0.031
MBR	5.4	0.565	0.453	0.199*	1.164	0.119
MDO	5.3	0.506	0.369	0.269*	1.032	0.000
MED	5.4	0.534	0.418	0.212*	1.095	0.000
MES	5.3	0.501	0.436	0.130	1.051	0.000
MMU	5.2	0.542	0.406	0.253*	1.114	0.000
MTM	6.0	0.591	0.506	0.140*	1.247	0.128
MTN	4.6	0.504	0.384	0.237*	1.006	0.000
MUN	5.7	0.569	0.470	0.177*	1.203	0.000
ONS	5.3	0.538	0.453	0.160*	1.109	0.094
PAR	5.4	0.487	0.369	0.237*	1.059	0.016
PIP	5.2	0.560	0.436	0.219*	1.148	0.000
SGP	6.1	0.518	0.429	0.173*	1.130	0.222
TAL	5.2	0.523	0.430	0.173*	1.075	0.000
TAM	5.1	0.446	0.324	0.276*	0.949	0.000
WHM	5.4	0.513	0.424	0.172*	1.084	0.000
*Mean*	*5*.*4(P = 0*.*001)*	*0*.*525(P = 0*.*001)*	*0*.*426(P = 0*.*656)*	*0*.*188(P = 0*.*001)*	*1*.*096*	*0*.*039*

Overall population divergence was greater in *A*. *atkinsiana* (*F*_*ST*_ = 0.190) than in *A*. *ancistrocarpa* (*F*_*ST*_ = 0.047). Isolation by distance (IBD) was present in *A*. *atkinsiana* (r = 0.343, *P* = 0.001), but not in *A*. *ancistrocarpa* (r = -0.058, *P* = 0.748).

Bayesian analyses for *A*. *atkinsiana* revealed a *K*_MAX_ of seven. Populations within the Hamersley Ranges and along the coast formed two distinct groups (Fig 1). Some populations situated at the edge of the range (TOM, CHIC, MTF, KUM and MARD) also formed distinct clusters, although the peripheral population at KAN was highly admixed. While most populations were largely assigned to a single cluster all populations had some degree of assignment to every cluster, the largest portions of which were to neighbouring clusters. In contrast, *A*. *ancistrocarpa* showed a lack of population structure, there was no clear plateau in the average DIC values, all individuals were highly admixed for all clusters, and there was no clear pattern to the clustering.

### Chloroplast haplotype diversity and phylogeographic structure

**Fig 3:**
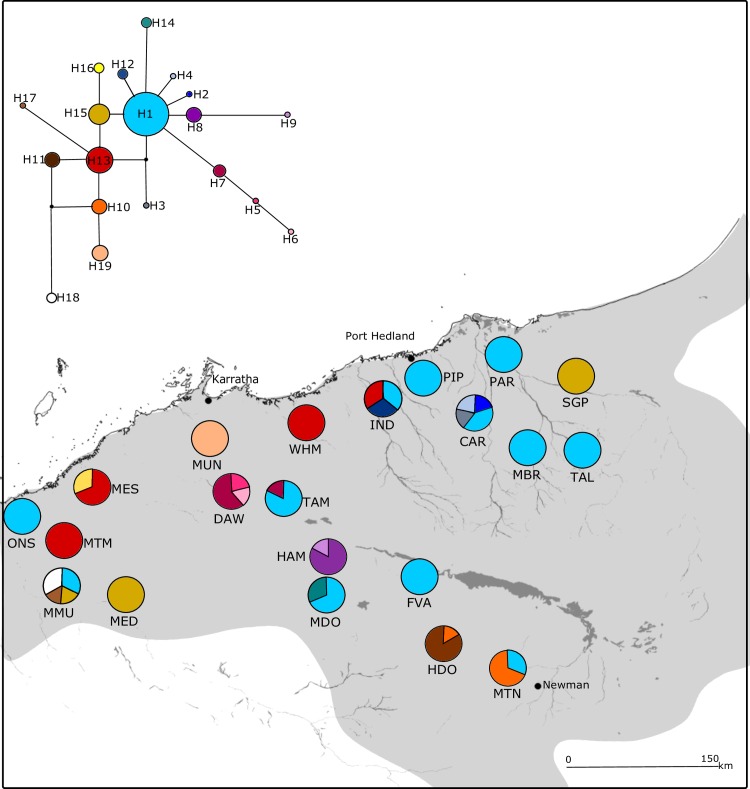
Geographical distribution of populations and chloroplast haplotypes for *Acacia atkinsiana*. The species distribution is shown in grey. All chloroplast haplotypes found in each population are shown with section sizes indicating the proportion of individuals with that haplotype. The haplotype network is shown in the upper left corner, each circle represents a distinct haplotype and its size indicates its relative frequency amongst all individuals. Branch length indicates the number of mutational steps between haplotypes and unobserved haplotypes are represented by black circles. Major water courses and the Fortescue floodplain are shown.

In *A*. *atkinsiana* the concatenated sequence was 2047bp with three polymorphic sites identified across 96 individuals (see S1 Table for GenBank accession numbers). In total, four haplotypes were identified, two (H1 and H2) were shared by 14 populations. The remaining two populations were fixed for one or other of the two rare haplotypes (H3, H4) (Fig 3). There did not seem to be a clear geographical pattern to the distribution of the common haplotypes that were spread across the entire species range. The other two haplotypes were restricted to different populations and geographic areas (Fig 3). Genetic diversity within populations (*H*_S_ = 0.142, *V*_*S*_ = 0.085) constituted less than a quarter of the total genetic diversity (*H*_T_ = 0.639, *V*_*T*_ = 0.474). Mean nucleotide diversity (π) was 0.007% and Tajima’s *D* did not deviate from the neutral model of evolution (Table 2).

**Table 2:** Diversity, differentiation and neutrality indices for chloroplast DNA data of *Acacia atkinsiana* and *A*. *ancistrocarpa*. Standard errors are given in parentheses.

Test/Measure	Statistic	*Acacia atkinsiana*	*Acacia ancistrocarpa*
Nucleotide diversity	*π*	0.007% (0.003%)	0.027%(0.009%)
Within population diversity (unordered)	*h*_*S*_	0.142 (0.056)	0.279 (0.073)
Within population diversity (ordered)	*v*_*S*_	0.085 (0.034)	0.193 (0.062)
Total diversity (unordered)	*h*_*T*_	0.639 (0.074)	0.807 (0.067)
Total diversity (ordered)	*v*_*T*_	0.474 (0.094)	0.671 (0.079)
Population Differentiation (unordered)	*G*_*ST*_	0.778 (0.094)	0.654 (0.086)
Population Differentiation (ordered)	*N*_*ST*_	0.821 (0.084)	0.713 (0.087)
Phylogeographic Structure	*N*_*ST*_*>G*_*ST*_	*P* = 0.08	*P* = 0.05
Neutrality test	*Tajima’s D*	0.043 (0.152), *P* = 0.883	0.038 (0.123), *P* = 0.787

In contrast, the concatenated sequence in *A*. *ancistrocarpa* was longer (2451bp) with more (21) polymorphic sites and haplotypes (19) across 124 individuals (see S1 Table for GenBank accession numbers). There was no clear pattern to the distribution of these haplotypes across populations (Fig 2): H1 was shared among 12 populations throughout the range, four populations along the coast contained H13, and H7 and H10 were shared by two neighbouring populations (DAW and TAM, and HDO and MTN, respectively). The other shared haplotype (H15) had a disjunct distribution and all other haplotypes arose in single populations only. Accordingly, compared to *A*. *atkinsiana*, more of the total diversity (*H*_T_ = 0.807, *V*_*T*_ = 0.671) was found within populations (*H*_S_ = 0.279, *V*_*S*_ = 0.193) and nucleotide diversity was also greater (π = 0.027%). Tajima’s *D* did not deviate from the neutral model of evolution (Table 2).

Genetic differentiation among populations was greater in *A*. *atkinsiana* (*N*_ST_ = 0.821, *G*_ST_ = 0.778) than in *A*. *ancistrocarpa* (*N*_ST_ = 0.713, *G*_ST_ = 0.654). *N*_ST_ was larger than *G*_ST_ for both species but was not significant (Table 2).

## Discussion

Both geographic range and distribution of populations influence patterns of genetic diversity and differentiation in two Pilbara acacias. In keeping with general theory on the relationship between genetic diversity and geographic range, the endemic, geographically restricted *A*. *atkinsiana* showed significantly lower levels of genetic variation within populations compared with the widespread *A*. *ancistrocarpa*. We also observed greater genetic divergence among populations of *A*. *atkinsiana* consistent with its patchily distributed populations, compared to the semi-continuous distribution of *A*. *ancistrocarpa*. These patterns were evident in both the nuclear and chloroplast genomes. There was little evidence of geographical influences on population genetic structure in *A*. *ancistrocarpa*, whereas *A*. *atkinsiana* exhibited contemporary genetic structure with differentiation of near-coastal populations from those in the ranges, and peripheral populations from those in the centre of the distribution. This highlights the importance of considering both geographic range and distribution of populations in predictions of genetic diversity and differentiation.

### Genetic diversity and range

Patterns of nuclear genetic diversity showed the restricted *A*. *atkinsiana* had significantly lower diversity than the widespread *A*. *ancistrocarpa*. While this is consistent with predictions, a number of studies have found restricted species to have levels of genetic variation similar to, or greater than, their widespread congeners [12, 13, 16, 20, 21] suggesting factors other than geographic range may have an influence on genetic variation. While reduced genetic variation in restricted or rare species is generally attributed to species persisting in small and isolated populations, an examination of two closely related rare species of *Lepidosperma* found lower genetic variation in the species with smaller and more disjunct populations but greater species range [79]. This finding implies distribution of populations may have a stronger influence in reducing genetic variation than geographic range. We suggest that the lower genetic variation in *A*.*atkinsiana* is likely also a consequence of its persistence in discrete populations compared to the larger semi-continuously distributed populations of *A*. *ancistrocarpa*.

The restricted *A*. *atkinsiana* also showed lower chloroplast nucleotide and haplotype diversity compared to *A*. *ancistrocarpa*. Patterns of haplotype diversity are not commonly investigated in geographically restricted species. These results demonstrate that range and distribution of populations have also influenced historical patterns of diversity and suggest these species may have maintained similar range and population configurations for a long time. A similar pattern of low haplotype diversity, along with low nuclear diversity, was observed in the rare *Eucalyptus caesia* that has a patchy distribution of populations due to being restricted to granite outcrops in a semi-arid environment [32].

Although the patterns of haplotype diversity differed in *A*. *atkinsiana* and *A*. *ancistrocarpa*, they support hypotheses of historical persistence in both species, with no signals of expansion. In general, both species have common haplotype(s) that are likely to be ancestral since they are distributed throughout the range, occupy central positions, and have multiple connections in the network. The less common haplotypes are located at the tips and tend to be geographically localised suggesting they have arisen from the common haplotype and dispersed locally [80, 81]. Exceptions were evident in *A*. *ancistrocarpa* with H15 being found at disjunct locations and H10 and H11 being geographically distant to genetically similar haplotypes. This may be the result of high levels of gene flow or, for H15, evidence of homoplasy. The high genetic variation and semi-continuous distribution of populations in *A*. *ancistrocarpa* would suggest gene flow, rather than homoplasy, as the most likely explanation for the distribution of H15.

### Genetic differentiation and distribution

There was greater population genetic structure in *A*. *atkinsiana* than *A*. *ancistrocarpa*, with higher genetic divergence and IBD. This is also most likely due to the difference in distribution between the two species, as this pattern is consistent with theoretical expectations based on differences in distributions of populations [5, 26–28]. The patchy distribution of *A*. *atkinsiana* populations would be expected to reduce gene flow across the species’ range in comparison with the semi-continuous distribution of populations in *A*. *ancistrocarpa*. However, our findings are in contrast to most studies that find little difference in genetic structure between localised and widespread species [6, 18, 19], noting that distribution of populations is not generally considered in many studies. Godt and Hamrick [22] suggest, based on an isolation-by-distance model, that gene flow would be reduced between distant populations of a widely distributed species compared to the more localised populations in a geographically restricted species. While the average distance between sampled populations of *A*. *ancistrocarpa* (263km) is indeed greater than *A*. *atkinsiana* (147km), genetic divergence is much lower (*F*_*ST*_ = 0.047 and 0.190 respectively) consistent with greater connectivity through gene flow among populations. Pollination in acacias tends to be facilitated by insects [54] and there is no reason to suggest different pollination mechanisms in the two species studied here. Thus the greater connectivity in *A*. *ancistrocarpa* is likely to be due to the influence of distribution of populations.

Extensive gene flow throughout the range of *A*. *ancistrocarpa* sampled here may be due to dispersal of seed by strong wind gusts or surface water flows associated with the passage of tropical depressions that are common across the region [82]. However, cyclonic wind and surface water flows would also disperse *A*. *atkinsiana* in a similar manner and therefore a similar pattern of population connectivity would be expected. Long distance seed dispersal may also be facilitated by birds since dispersal of seeds by emus across large areas is common and has been recorded for other acacia species in Western Australia [83], and suggested for *Eucalyptus camaldulensis* in the Pilbara [49]. Neither *Acacia* species studied here has any obvious adaptations for dispersal, and would equally likely have seed dispersed by emus. However, emus are more common on the plains and grassy flats, where *A*. *ancistrocarpa* is prevalent, than in the upland ranges where *A*. *atkinsiana* is found. Over time, therefore, bird mediated seed dispersal is likely to have been greater in *A*. *ancistrocarpa* than in *A*. *atkinsiana*.

Phylogeographic structure in the chloroplast genome was also consistent with expectations based on population size and distribution. In small isolated populations, new mutations are more likely to be lost through drift than in large populations, but if they are retained and become more prevalent, they are unlikely to spread throughout the species range, remaining in localised areas [26]. This is the pattern found in the patchily distributed *A*. *atkinsiana* where there were four haplotypes, with two common haplotypes spread throughout the range, while the two rare haplotypes were highly localised and detected in only one or two populations. In contrast, new mutations will disperse more rapidly and over a greater area in large connected populations maintaining greater diversity and less differentiation [26]. This pattern was evident in the semi-continuous *A*. *ancistrocarpa*, where the most common haplotype was found throughout the range, although not present in all populations, and several other haplotypes were shared across populations, some with large geographic disjunctions, along with many haplotypes with localised distributions. Thus, distribution and size of populations of these two acacia species appears to have had a demonstrable effect on the amount and distribution of chloroplast genetic variation.

The distribution of the common chloroplast haplotypes in *A*. *atkinsiana* was largely independent of the geographical location of populations. This could be due to the accumulation and retention of ancestral polymorphism [81, 84] or historic gene flow via seed dispersal across the species range. However, the high nuclear genetic divergence between populations does not support high levels of seed dispersal. Lack of phylogeographic structure associated with retention of polymorphism is consistent with historical persistence [81] and such retention of ancestral polymorphism has also been observed in species with localised distributions in arid ecosystems [79, 85].

### Biotic patterns in the Pilbara

Given the lack of genetic studies on plants in the Pilbara, it is opportune to consider the patterns of genetic diversity and differentiation observed in these two species in connection with the landscape they occupy. Previous studies of animals endemic to the Pilbara have shown an affiliation between genetic structure and the geology and physiogeography of the region [41–45]. Genetic structure was limited in the few other Pilbara plant studies conducted [49–51] but there was evidence of the Hamersley Range being a refugium in *E*. *leucophloia* [51] and persistance through glacial cycles in Pilbara and inland ranges in *C*. *glaucophylla* [50]. In the two acacia species studied here, contemporary population genetic structure seems to have been largely obscured by high levels of gene flow in *A*. *ancistrocarpa*, while in *A*. *atkinsiana* it seemed to be influenced by geographical features. In two regions, Hamersley/Chichester Ranges and near-coastal areas, multiple populations were largely assigned to the same genetic cluster, indicating that frequent gene flow occurs among these populations. Populations located at the periphery of the species range were mostly assigned to distinct clusters suggesting greater isolation, with reduced gene flow and enhanced genetic drift leading to genetic divergence [86]. Natural selection is also likely to play an important role in driving genetic divergence in peripheral populations as environments at the species boundary are often different to central environments [86]. However, the species range is not large in *A*. *atkinsiana* and there are no obvious environmental differences in the populations on the periphery of the Hamersley and Chichester Ranges. The population at CHIC may be further isolated by the Fortescue River that separates it from the other populations within the Hamersley Range. Populations found in the Fortescue River floodplain, namely MTF and KAN, while further away from CHIC than some of the Hamersley populations, have reasonable proportions of admixture with CHIC, suggesting gene flow occurs more frequently along the river floodplain than across it. However, because the Fortescue is a seasonal river, the river floodplain is unlikely to present a complete barrier to dispersal. All populations exhibited some degree of admixture with most clusters, and this pattern supports an explanation of IBD superimposed on a landscape where partial barriers to gene flow are present, generating large clusters made up of several neighbouring populations, with genetically distinct peripheral populations and a degree of admixture throughout the range.

The evidence of greater connectivity among the Hamersley and Chichester Range populations of *A*. *atkinsiana* is consistent with hypotheses of ranges providing heterogeneous conditions that are most suited to historical persistence [52]. Alternatively, this pattern could be a consequence of the species distribution being centred primarily on the ranges, providing greater opportunities for gene flow. The connectivity among the near-coastal populations is also consistent with a hypothesis of thermal buffering of coastal areas providing greater opportunities for historical persistence [52]. Previous phylogeographic studies in the Pilbara have focused on animal species with limited dispersal capabilities [41, 42, 45] where patterns appear to be strongly influenced by environment. The absence of strong geographical influences on population genetic structure in both *A*. *atkinsiana* and *A*. *ancistrocarpa* may be due to greater connectivity among populations than in previously studied Pilbara animal systems.

### Conclusions

Our results indicate that geographic range (localised versus widespread) and the distribution of populations (patchy vs semi-continuous) have had significant impacts on the amount of genetic variation within populations and the population genetic structure of two Pilbara acacias. While genetic diversity was consistent with predictions based on geographic range, as generally observed in comparisons of geographically restricted and widespread species, genetic differentiation was consistent with population distribution with the patchily distributed species showing greater differentiation than the more continuously distributed species. The contrasting patterns observed here demonstrate that conservation approaches for species management and ecological restoration need to consider the distribution of populations in geographically restricted species. For example, our study provides important information about optimal seed sourcing for mine site rehabilitation, a common activity in the iron ore rich Pilbara region. Identification of appropriate seed sources, through knowledge of patterns of genetic diversity and differentiation, that aim to optimise genetic diversity while minimising risk of outbreeding depression [87] is an important component of effective restoration practice [88]. Our results suggest, that in contrast to the widespread *A*. *ancistrocarpa* where broad provenance collection zones are appropriate, seed collections for the restricted *A*. *atkinsiana* should be sourced more locally according to the patterns of relatively high genetic differentiations.

## Supporting Information

S1 Table GenBank accession numbers for *Acacia atkinsiana* and *A*. *ancistrocarpa* chloroplast intergenic spacer regions. (DOCX)
