# Supplementary material for: Contrasting Influences of Geographic Range and Distribution of Populations on Patterns of Genetic Diversity in Two Sympatric Pilbara Acacias
Source: PLoS One. 2016 Oct 21;11(10):e0163995. doi: 10.1371/journal.pone.0163995 (PMC5074490; doi:10.1371/journal.pone.0163995)
Supplement: S1 Table — (DOCX) [file pone.0163995.s001.docx]

**S1 Table**: GenBank Accession Numbers for *Acacia atkinsiana* and *A. ancistrocarpa* chloroplast intergenic spacer regions and haplotypes.

|  | **GenBank Accession Number** | | |
| --- | --- | --- | --- |
| **Species/Haplotype** | **atpF D-loop** | **trnS-trnG** | **trnV-ndhC** |
| ***A.atkinsiana*** |  |  |  |
| H1 | KT693989 | KT693991 | KT693994 |
| H2 | KT693989 | KT693991 | KT693993 |
| H3 | KT693989 | KT693992 | KT693994 |
| H4 | KT693990 | KT693991 | KT693994 |
| ***A.ancistrocarpa*** |  |  |  |
| H1 | KT693995 | KT694008 | KT693997 |
| H2 | KT693995 | KT694007 | KT693997 |
| H3 | KT693995 | KT694009 | KT693997 |
| H4 | KT693995 | KT694010 | KT693997 |
| H5 | KT693995 | KT694008 | KT693998 |
| H6 | KT693995 | KT694008 | KT693999 |
| H7 | KT693995 | KT694008 | KT694000 |
| H8 | KT693995 | KT694008 | KT694001 |
| H9 | KT693995 | KT694011 | KT694001 |
| H10 | KT693995 | KT694015 | KT693997 |
| H11 | KT693995 | KT694012 | KT694002 |
| H12 | KT693996 | KT694008 | KT693997 |
| H13 | KT693995 | KT694012 | KT693997 |
| H14 | KT693995 | KT694008 | KT694003 |
| H15 | KT693995 | KT694013 | KT693997 |
| H16 | KT693995 | KT694013 | KT694004 |
| H17 | KT693995 | KT694014 | KT693997 |
| H18 | KT693995 | KT694015 | KT694005 |
| H19 | KT693995 | KT694015 | KT694006 |
